# Ectoine degradation pathway in halotolerant methylotrophs

**DOI:** 10.1371/journal.pone.0232244

**Published:** 2020-04-30

**Authors:** Aleksander S. Reshetnikov, Olga N. Rozova, Yuri A. Trotsenko, Sergey Yu. But, Valentina N. Khmelenina, Ildar I. Mustakhimov

**Affiliations:** Federal Research Center «Pushchino Scientific Center for Biological Research of the Russian Academy of Sciences», G.K. Skryabin Institute of Biochemistry and Physiology of Microorganisms of the Russian Academy of Sciences, Pushchino, Russian Federation; University of Münster, GERMANY

## Abstract

**Background:**

Microorganisms living in saline environments are forced to regulate turgor via the synthesis of organic osmoprotective compounds. Microbial adaptation to fluctuations in external salinity includes degradation of compatible solutes. Here we have examined the biochemical pathway of degradation of the cyclic imino acid ectoine, the major osmoprotector in halotolerant methane-utilizing bacteria.

**Methods:**

The BLAST search of the genes involved in ectoine degradation in the halotolerant methanotroph *Methylotuvimicrobium alcaliphilum* 20Z was performed with the reference sequences of *Halomonas elongata*. The genes for the key enzymes of the pathway were disrupted by insertion mutagenesis and the cellular metabolites in the methanol extracts of mutant cells were analyzed by HPLC. The *doeA* gene from *Mm*. *alcaliphilum* 20Z was heterologously expressed in *Escherichia coli* to identify the product of ectoine hydrolysis catalyzed by ectoine hydrolase DoeA.

**Results:**

We have shown that the halotolerant methanotroph *Mm*. *alcaliphilum* 20Z possesses the *doeBDAC* gene cluster coding for putative ectoine hydrolase (DoeA), Nα-acetyl-L-2,4-diaminobutyrate deacetylase (DoeB), diaminobutyrate transaminase (DoeD) and aspartate-semialdehyde dehydrogenase (DoeC). The deletion of the *doeA* gene resulted in accumulation of the higher level of ectoine compared to the wild type strain. Nγ-acetyl-L-2,4-diaminobutyrate (Nγ-acetyl-DAB), a substrate for ectoine synthase, was found in the cytoplasm of the wild type strain. Nα-acetyl-L-2,4-diaminobutyrate (Nα-acetyl-DAB), a substrate for the DoeB enzyme, appeared in the cells as a result of exposure of the *doeB* mutant to low osmotic pressure. The genes for the enzymes involved in ectoine degradation were found in all aerobic methylotrophs capable of ectoine biosynthesis. These results provide the first evidence for the *in vivo* operation of the ectoine degradation pathway in methanotrophs and thus expand our understanding of the regulation mechanisms of bacterial osmoadaptation.

**Conclusions:**

During adaptation to the changes in external osmolarity, halophilic and halotolerant methylotrophs cleave ectoine, thereby entering the carbon and nitrogen of the compatible solute to the central metabolic pathways. The biochemical route of ectoine degradation in the halotolerant methanotroph *Mm*. *alcaliphilum* 20Z is similar to that in heterotrophic halophiles. We have shown that ectoine hydrolase DoeA in this methanotroph hydrolyzes ectoine with the formation of the only isomer: Nα-acetyl-DAB. All aerobic methylotrophs capable of ectoine biosynthesis harbor the genetic determinants for ectoine degradation.

## Introduction

Microorganisms living in saline environments are enforced to synthesize highly water-soluble organic compounds and accumulate them at concentrations sufficient to maintain cell turgor. Ectoine (1,4,5,6,tetra-2-methyl-4-pyrimidinecarboxylic acid) and its derivative hydroxyectoine are compatible solutes in many halophilic bacteria and some Archaea [1–6]. In addition to balancing the osmotic pressure between the cytoplasm and its surroundings, ectoines can exert beneficial effects on microbial metabolism as they stabilize protein folding and protect whole cells against various stresses such as UV radiation, freezing, drying, and high temperatures [7–9]. Ectoine accumulation by *de novo* synthesis is an essential trait of halotolerant methylotrophs, the bacteria utilizing methane or its oxidized or substituted derivatives as carbon and energy sources [10, 11]. The aerobic methanotroph *Mm*. *alcaliphilum* 20Z isolated from a Siberian soda lake is a halotolerant bacterium able to grow at salinity up to 10% NaCl [10]. It synthesizes ectoine, glutamate and sucrose as the main compatible solutes. The genes encoding the enzymes for ectoine biosynthesis are localized in the *ectABC-ask* operon transcribed from two σ^70^-dependent promoters under the control of the MarR-family regulator EctR [12]. The biosynthesis of ectoine can also be regulated at the level of enzyme activities [13].

Fluctuation in salinity is a ubiquitous stress factor in many natural habitats of microorganisms. When exposed to low external osmolarity, microbial cells can modulate turgor by the expulsion of previously accumulated compatible solutes through the transient opening of mechanosensitive channels [14].

Many microorganisms can use the released compatible solutes as osmostress protectants or as nutrients [15]. In the Gram-negative bacteria *Chromohalobacter salexigens*, *Sinorhizobium meliloti* and *Ruegeria pomeroyi* DSS-3, utilization of ectoine and hydroxyectoine has been found to proceed via the pathway where the key constituents are the enzymes (EutABCDE; *eut*: **e**ctoine **ut**ilization) [16–18] catalyzing the conversion of hydroxyectoine to ectoine (EutABC) and ectoine decomposition to acetate and diaminobutyrate (EutDE). It also includes the genes for specific diaminobutyrate aminotransferase and aspartate semialdehyde dehydrogenase catalyzing the synthesis of aspartate. The genes for these enzymes are co-transcribed with the genes for transport systems: either a binding-protein-dependent ABC (EhuABCD; *ehu*: **e**ctoine-**h**ydroxyectoine **u**ptake) transporter or a 5-hydroxyectoine/ectoine-specific TRAP transporter [18–21]. In the salt-tolerant bacterium *Halomonas elongata*, a gene cluster related to those previously involved in ectoine degradation in *S*. *meliloti* and *R*. *pomeroy*i DSS-3 has been identified [6]. Four enzymes of the pathway encoded by the *doeAB* and *doeCD* genes (**doe: d**egradation **o**f **e**ctoine) catalyze ectoine hydrolysis to N-acetyl-DAB and further deacetylation of the latter with the formation of diaminobutyrate and acetate [6]. DAB can either flow off to aspartate or re-enter the ectoine synthesis pathway, forming a cycle of ectoine synthesis and degradation.

However, the ectoine degradation ability in methylotrophic ectoine producers has not been investigated thus far. In this paper we have shown, on the basis of genomic and mutational analysis, that the ectoine degradation pathway is functional in the halotolerant methanotroph *Mm*. *alcaliphilum* 20Z.

## Materials and methods

### Bacteria and growth conditions

*Methylotuvimicrobium alcaliphilum* 20Z (earlier *Methylomicrobium*) (NCIMB 14124^T^ = VKM B-2133^T^) was routinely cultivated at 30°C in a methane–air atmosphere (1 : 1) or in the presence of 0.3% methanol (v/v) in the nitrate-containing 2×P medium supplemented with 0.1 M NaHCO_3_ and different amounts of NaCl as described [10]. *Escherichia coli* S17-1 and Rosetta (DE3) obtained from Stratagene (USA) were routinely grown at 37°C in the LB medium [22]. Gentamicin (10 μg/ml) or kanamycin (100 μg/ml) was added to the growth medium if required.

### Cloning and expression of the DoeA and DoeB enzymes in *E*. *coli*

The DNA from *Mm*. *alcaliphilum* was prepared with a ZymoResearch Fungal/Bacterial DNA MiniPrep™ kit (Irvine, USA). The genes coding for the putative DoeA (*doeA*, MEALZ_3978) and DoeB (*doeB*, MEALZ_3976) were amplified using the primers listed in S1 Table. The PCR-products were treated with endonucleases NdeI and HindIII and ligated in expression vector pET22b opened at the respective restriction sites. The resulting plasmids were transferred into *E*. *coli* Rosetta (DE3) and protein expression was induced by 0.5 mM isopropyl β-D-1-thiogalactopyranoside (IPTG, Sigma-Aldrich, USA) added in the logarithmic growth phase (OD_600_ = 0.6–0.8). After 18-h incubation at 18°C, cells were collected by centrifugation (5,000 *g* for 30 min, 4°C) and disrupted in an ultrasonic disintegrator (Misonix, USA) with 1-min cooling in ice after each 10-s sonication. The suspension was centrifuged for 30 min at 11,000 *g* and 4°C and the soluble fraction was analyzed by SDS-polyacrylamide gel electrophoresis.

### The phylogenetic analysis

The nucleotide sequences of the *doe* genes were obtained from the NCBI database (http://www.ncbi.nlm.nih.gov) by BLAST searches. The alignments of amino acid sequences were generated with ClustalW of the MEGA 6 program [23]. The minor corrections in sequence alignments were made manually. Phylogenetic analysis was carried out using the MEGA 6 program and the Maximum Likelihood model.

### Mutant generation

Insertions into the *doeA* and *doeB* genes were introduced using the suicide vector pCM184 [24] as described [12]. Briefly, the 3′- and 5′-fragments of each gene to be mutated were PCR-amplified using the appropriate primers (S1 and S2 Tables) and inserted into pCM184 upstream and downstream of the kanamycin (Km) resistance gene. The cells of *E*. *coli* S17-1 [25] were transformed by the resultant plasmids, and the donor strains were obtained and mated with the wild-type *Mm*. *alcaliphilum* 20Z. Mutants were selected on the plates with a mineral medium containing 0.5% methanol (v/v) and 100 μg/ml kanamycin. Double-crossover mutants were identified by diagnostic PCR tests. The *Mm*. *alcaliphilum* strain Δ*ectBC* deficient in two enzymes for ectoine biosynthesis, i.e., diaminobutyrate aminotransferase and ectoine synthase, was obtained as described [26].

### Homologous overexpression of the *ectABC*-operon

The *ectABC* gene cluster was amplified from *Mm*. *alcaliphilum* DNA and overexpressed in the wild type strain and in the strain with disrupted *doeA* by using the previously designed vector pMHA200_Pmxa_cat [27]. The catalase-encoding *cat* gene was replaced by the *ectABC* cluster inserted between the SacI and VspI sites. In the resultant construct pMHA200_Pmxa_ectABC, the kanamycin resistance gene was replaced by the gentamicin resistance gene amplified from the vector p34S-Gm employing the restriction sites for PstI (S1 and S2 Tables). *E*. *coli* S17-1 cells were transformed by the resultant plasmid pMHA300_Pmxa_ectABC and transferred into *Mm*. *alcaliphilum* strains by conjugation. Conjugants were selected on the plates with the mineral medium P containing 10 μg/ml Gm (if the cells of the wild-type strain were transformed) or 100 μg/ml Km and 10 μg/ml Gm (if the cells of the Δ*doeA* mutant were transformed). The presence of the pMHA300_Pmxa_ectABC plasmid in the cells of *Mm*. *alcaliphilum* 20Z was identified by diagnostic PCR tests.

*Expression of the doeA gene in E*. *coli*. To identify the product of DoeA reaction, the *doeA* gene was expressed in *E*. *coli* XL1-Blue from the P_*lac*_ promoter. A PCR fragment containing the *doeA* sequence was amplified from the *Mm*. *alcaliphilum* DNA using primers DoeA_f and DoeA_r (S1 and S2 Tables) and cloned in the low-copy-plasmid pHSG575 opened by endonucleases EcoRI and HindIII [28]. *E*. *coli* cells transformed by the pHSG575_doeA plasmid and those without the plasmid were grown in the mineral medium M9 containing glucose, 3% NaCl and 4 mM ectoine. 10 ml of each culture was harvested by centrifugation: (i) *E*. *coli* harboring the plasmid pHSG575_doeA grown without IPTG; (ii) *E*. *coli* harboring the plasmid pHSG575_doeA grown in the presence of 0.5 mM IPTG and (iii) *E*. *coli* not harboring the plasmid. The methanol extract from the cells of each culture was analyzed by HPLC to identify intracellular metabolites. The Nα-acetyl-DAB and Nγ-acetyl-DAB standards were obtained by the alkaline hydrolysis of pure ectoine [29].

*Analytical methods*. The methanol/chloroform extraction procedure was used to isolate organic solutes from cells as described [23]. The excretion of ectoine into the growth medium was checked by analyzing freeze-dried culture liquid re-dissolved in an acetonitrile/water mixture (70:30 v/v). Ectoine concentration was measured by isocratic HPLC (LC-20 Prominence, Shimadzu) in a Reprosil 100 NH2 column (4×150 mm, 3 μm) using acetonitrile/water (70:30 v/v) as the mobile phase and UV detection at 210 nm. The commercially available ectoine (Sigma) was used for the standard curve preparation. Quantification of glutamate was performed with a glutamate assay kit (Sigma) according to the manufacturer's instructions. Sucrose was analyzed by HPLC (LC-20 Prominence, Shimadzu) with a refractive index (RI) detector (RID-20A, Shimadzu) using a ReproGel H+ column (length, 300 mm; particle size, 7.8 mm). The samples were separated at 50 ⁰C using an isocratic elution program at a flow rate of 0.5 ml/min; mobile phase was 1 mM H_**2**_SO_**4**_. The commercially available sucrose (Sigma) was used for standard curve preparation. Nα-acetyl-DAB and Nγ-acetyl-DAB were identified by HPLC (LC-20 Prominence, Shimadzu) in a Reprosil OPA column (4.6×150 mm, 3 μm) with o-phthalaldehyde (OPA) pre-column derivatization and detection at 330 nm. The samples were separated at 25 ⁰C with a gradient elution program at a flow rate of 1 ml/min. The mobile phase was potassium phosphate buffer (pH 7.2) (solvent A) or potassium phosphate buffer–methanol–acetonitrile mixture (50:35:15, v/v) (solvent B). The gradient elution program was as follows: 100–0% A (0–40 min), 100% B (40–50 min), 100–0% B (55–60 min) and 100% A (60–75 min).

### DNA manipulations

Plasmid isolation, digestion by restriction enzymes, agarose gel electrophoresis, ligation and transformation of *E*. *coli* cells were performed according to [22]. Restriction enzymes, T4 DNA ligase, Pfu DNA polymerase and dNTPs were purchased from Thermo Scientific (USA).

### Osmotic down-shock experiments

100 ml of the Δ*doeB* culture grown exponentially in the presence of 6% NaCl was diluted four times by the mineral medium P without NaCl and incubated under the optimal temperature and aeration. The cells were harvested by centrifugation from 100 ml of each culture: (i) grown at 6% NaCl but not exposed to down shock; (ii) diluted and incubated for 40 and (iii) for 90 min. The intracellular metabolites were identified in methanol extracts by HPLC.

## Results

### Identification of the genes encoding ectoine degradation enzymes

**Fig 1:**
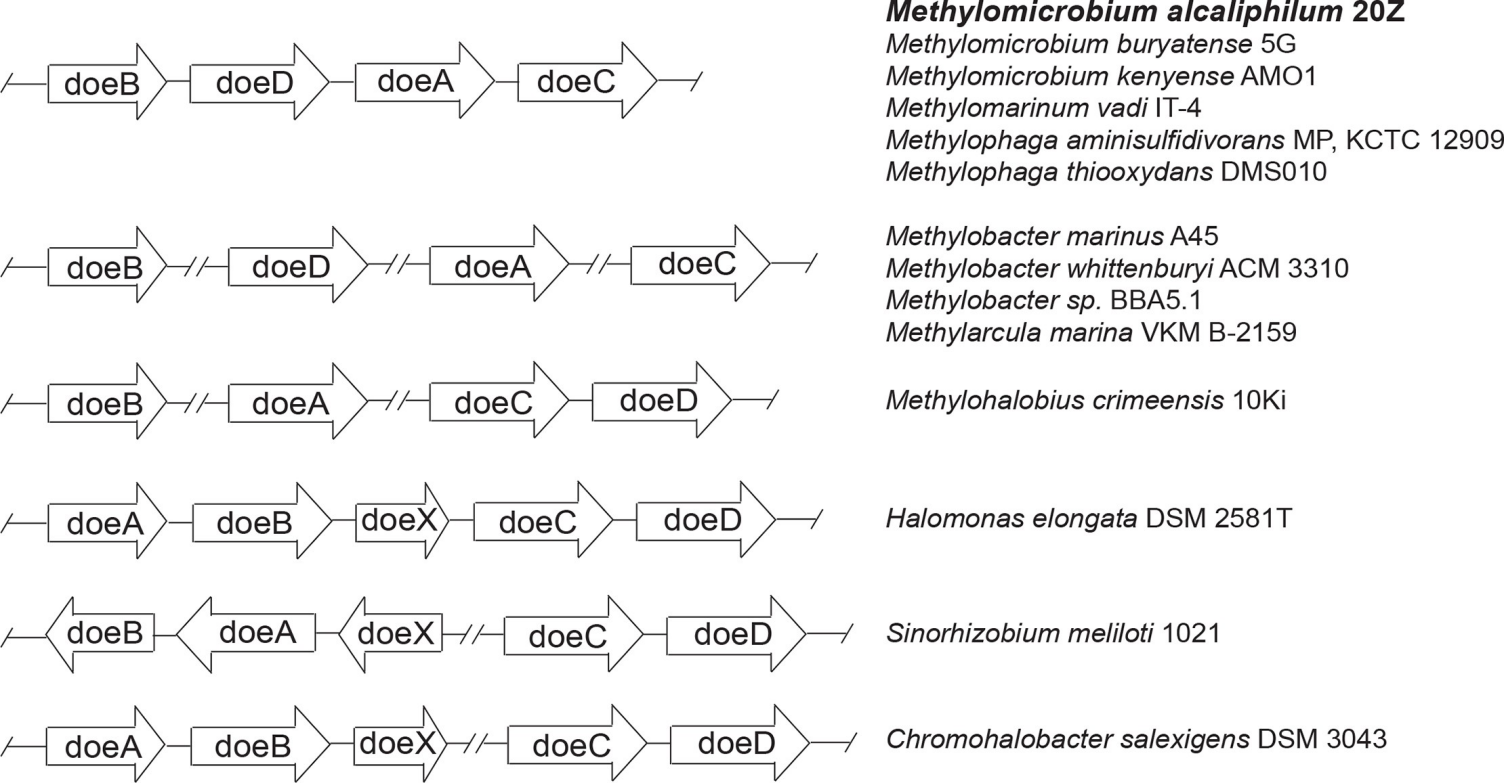
The arrangement of ectoine degradation genes in methylotrophic and some heterotrophic bacteria. Heterotrophic bacteria with the established ectoine degradation pathway are presented. *doeA*, ectoine hydrolase; *doeB*, Na-acetyl-L-2,4-diaminobutyric acid deacetylase; *doeX*, AsnC/Lrp-like DNA-binding protein; *doeC*, aspartate-semialdehyde dehydrogenase; *doeD*, L-2,4-diaminobutyrate transaminase. Only ectoine degradation genes, without transporter genes and regulatory proteins, are indicated for the heterotrophic bacteria, since they are absent in the considered clusters of methylotrophs.

The genes potentially involved in ectoine degradation were identified by comparing the genomes of *Mm*. *alcaliphilum* 20Z and *H*. *elongata*. In the methanotroph, four open reading frames showing moderate similarities to the *doeA*, *doeB*, *doeС* and *doeD* genes coding for the enzymes of ectoine degradation in *H*. *elongata* (42, 22, 36 and 49% identities) were adjacently located (Fig 1, S3 Table). These genes are oriented in the same direction, making up the *doeBDAC* cluster. The organization of the cluster differs from the *H*. *elongata* ectoine degradation operon, which additionally codes for the AsnC/Lrp-like DNA-binding protein, the MocR/GabR-type transcriptional regulator EnuR, and the two enzymes involved in the conversion of hydroxyectoine into ectoine [6, 18].

**Fig 2:**
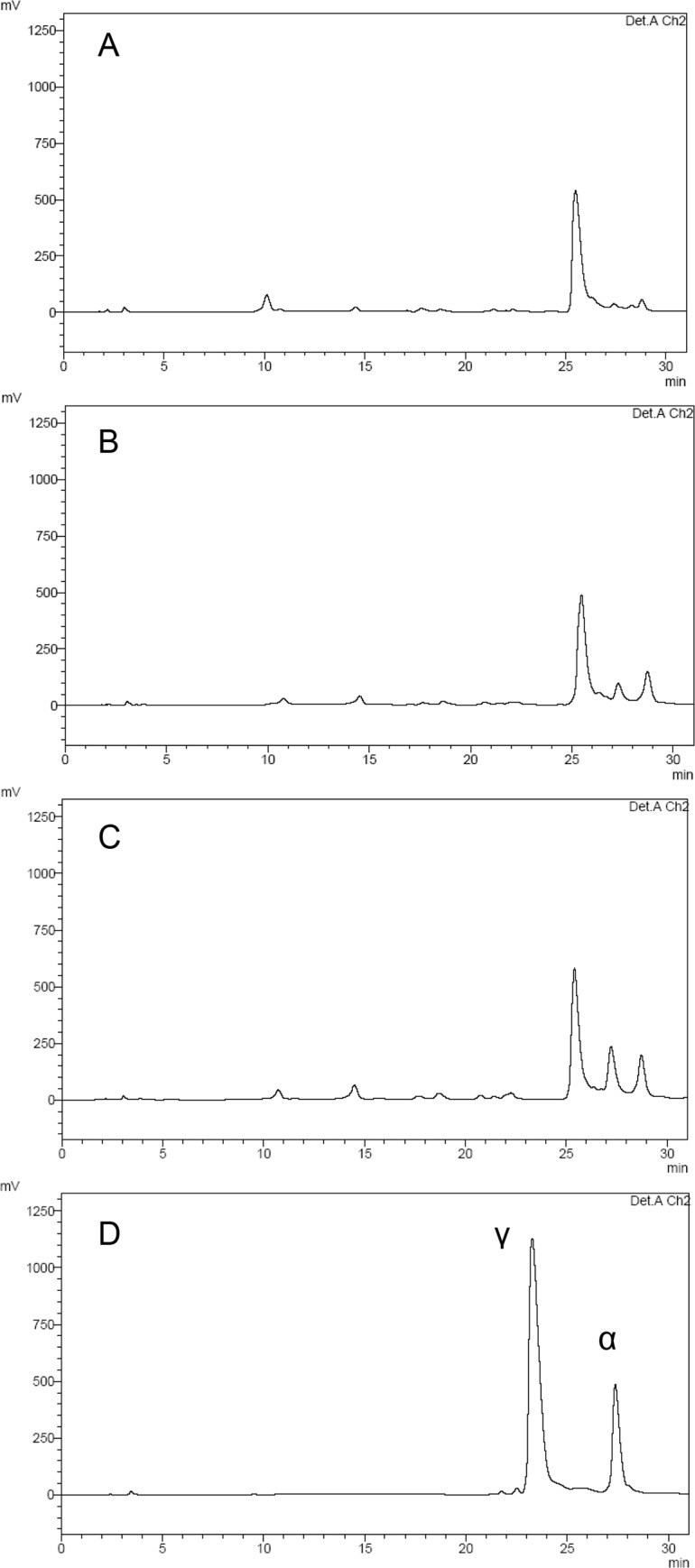
The chromatograms of methanol extracts from *E*. *coli* cells transformed by plasmids pHSG575 (A) and pHSG575_doeA carrying the *doeA* gene from *Mm*. *alcaliphilum* (B, C). *E*. *coli* cells were grown in the presence of 3% NaCl and 4 mM ectoine. B, cells grown without IPTG; C, cells grown in the presence of 0.5 mM IPTG; D, standards of Nγ-acetyl-DAB and Nα-acetyl-DAB obtained by the alkaline hydrolysis of pure ectoine [29]. Intracellular metabolites were analyzed by HPLC.

We failed to obtain functional recombinant DoeA and DoeB enzymes from *Mm*. *alcaliphilum* 20Z by heterologous expression of the *doeA* or *doeB* genes in *E*. *coli*, since *E*. *coli* Rosetta (DE3) transformed by the pET22b_doeA or pET22b_doeB plasmids synthesized mostly insoluble protein forms. The variation of cultivation temperature, the replacement of the T7 promoter by a weaker arabinose promoter, or the use of the pHUE plasmid for expression of proteins with an additional ubiquitin peptide was ineffective. Nevertheless, Nα-acetyl-DAB was found in the *E*. *coli* cells transformed by the low-copy plasmid pHSG575_doeA and grown in the presence of 4 mM ectoine and 3% NaCl (Fig 2). On the contrary, two isomers, Nα- and Nγ-acetyl-DAB, have been found in *E*. *coli* expressing *doeA* from *H*. *elongata* [6]. Thus, it might be supposed that DoeA from *Mm*. *alcaliphilum* 20Z catalyzes a more specific reaction of ectoine hydrolysis, generating Nα-acetyl-DAB but not forming Nγ-acetyl-DAB.

### Phenotypic characterization of the Δ*doeA* and Δ*doeB* mutants

To check the function of the putative *doeA* gene in *Mm*. *alcaliphilum* 20Z, this gene was disrupted by insertion of a kanamycin cassette using the suicidal vector pCM184. The growth rate of the knockouted strain was not changed, but the mutant accumulated a higher level of ectoine compared to the wild-type strain when grown at 1 or 3% NaCl (Table 1).

**Table 1:** The intracellular accumulation of ectoine and glutamate (mg/g wet biomass) in different strains of *Mm*. *alcaliphilum* 20Z.

Strain	1% NaCl		3% NaCl	
	Ectoine	Glutamate	Ectoine	Glutamate
WT	0.11 ± 0.045	0.91 ± 0.08	8.9 ± 0.18	5.1 ± 0.38
WT pMHA300_Pmxa_ectABC	0.58 ± 0.048	1.09 ± 0.06	13.8 ± 0.24	4.92 ± 0.46
Δ*doeA*	0.13 ± 0.034	0.95 ± 0.12	9.1 ± 0.16	6.19 ± 0.52
Δ*doeA* pMHA300_Pmxa_ectABC	1.55 ± 0.05	0.98 ± 0.065	14.8 ± 0.18	5.32 ± 0.35

The pMHA300_Pmxa_ectABC plasmid carrying the *ectABC* gene cluster under the control of the constitutive methanol dehydrogenase promoter Pmxa was introduced into both the wild type strain *Mm*. *alcaliphilum* and its Δ*doeA* mutant. Such transformation was performed in order to reduce the contribution of EctR to the transcriptional regulation of expression of the *ect*-genes [12]. During the growth at a low salinity (1% NaCl), the cells of the wild type strain transformed by the pMHA300_Pmxa_ectABC plasmid accumulated fivefold more ectoine compared to the strain without this plasmid. The Δ*doeA* mutant transformed by the same plasmid had a threefold higher ectoine level than the wild type strain (Table 1). The differences in ectoine contents between the wild type strain and the Δ*doeA* mutant growing at a salinity of 3% were less pronounced. These data implied the participation of DoeA in the regulation of intracellular ectoine level. In both strains, the intracellular glutamate content gradually increased in response to an increase in salinity of the medium, but intracellular glutamate levels did not show any noticeable dependence on strain modification.

**Fig 3:**
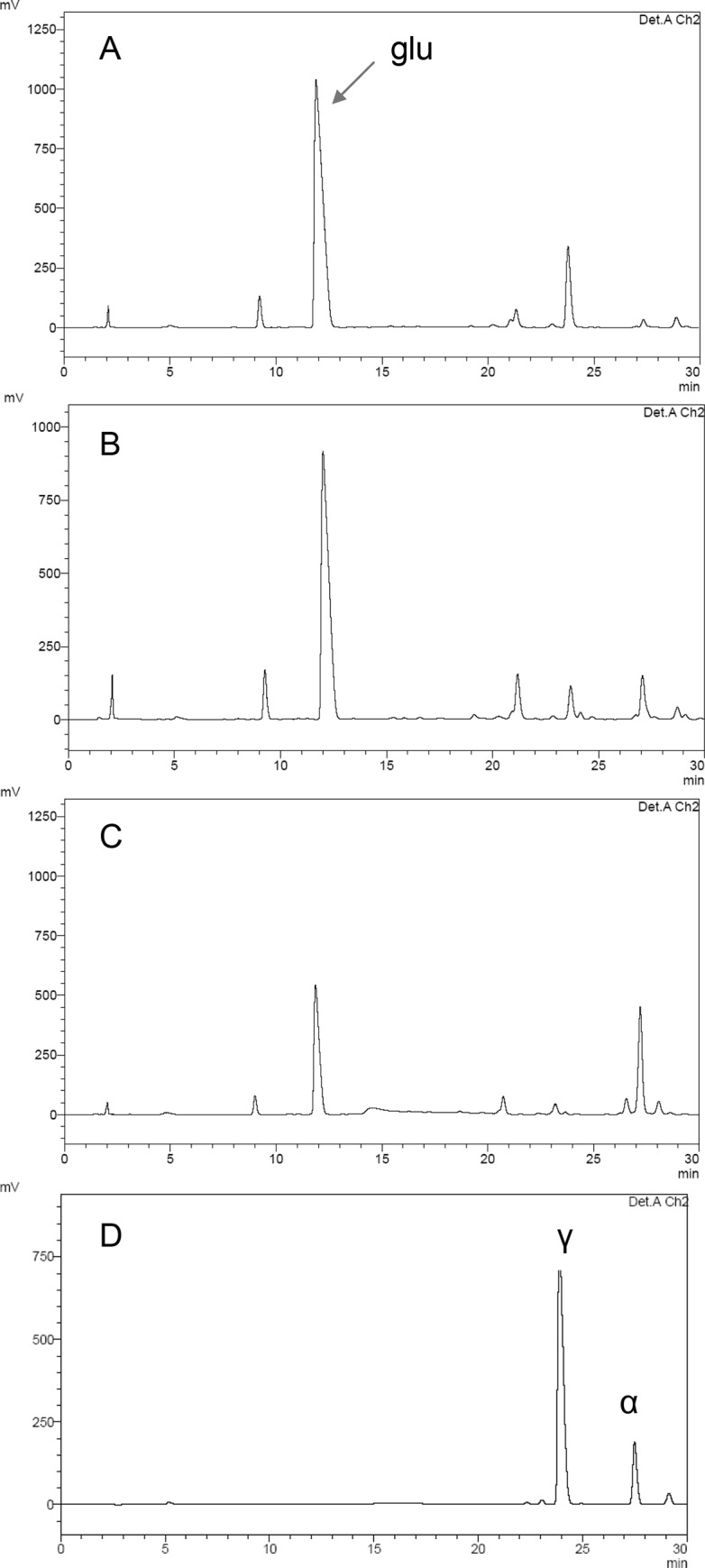
The chromatograms of intracellular solutes in the ΔdoeB mutant of *Mm*. *alcaliphilum* in the Reprosil OPA column with o-phthalaldehyde (OPA) precolumn derivatization. The culture was grown under methane in a mineral salt medium in the presence 6% NaCl (A), diluted with the medium without NaCl to a final concentration of 1.5% NaCl (B, C) and incubated for 40 min (B) or 90 min (C) under the optimal growth conditions. D, the standards of Nγ-acetyl-DAB and Nα-acetyl-DAB obtained by the alkaline hydrolysis of pure ectoine [29].

The strain impaired in the *doeB* gene (Δ*doeB*) was obtained by the insertion of kanamycin cassette. Methanol extracts from cells of the Δ*doeB* strain grown at 6% NaCl had a peak corresponding to Nγ-acetyl-DAB. However, the peak of Nγ-acetyl-DAB gradually disappeared after osmotic down-shock as a result of fourfold dilution of the culture with the growth medium without NaCl and 40-min incubation under the optimal growth conditions (Fig 3). The peak of Nα-acetyl-DAB (a substrate for DoeB) was detected after further 50-min incubation in the diluted medium (Fig 3). Therefore, we assume that the osmotic down-shock induced ectoine cleavage into Nα-acetyl-DAB and this isomer is a substrate for DoeB enzyme. In the wild type cells, the peak of Nα-acetyl-DAB was found but did not increase substantially during exposure in the diluted medium (S1 Fig).

### Genomic analysis of the ectoine degradation pathway in methylotrophs

The homologs of ectoine degradation genes were revealed in all halophilic/halotolerant aerobic methylotrophs capable of ectoine biosynthesis (Fig 1). In most methylotrophs, these genes are organized in the *doeBDAC* cluster. The exception is *Methylohalobius crimeensis* possessing the *doeCD* cluster and individual *doeA* and *doeB* genes, as well as methanotrophic representatives of the genus *Methylobacter* (*M*. *marinus*, *M*. *whittenburyi* and *Methylobacter* sp. BBA5.1), where these genes are scattered on chromosomes (Fig 1). The differences in the genetic organization correlated with the level of sequence divergence of the proteins. For example, the proposed enzymes from all non-methanotrophic methylotrophs of the genus *Methylophaga* encoded by the *doeBDAC* cluster are most similar to the *Mm*. *alcaliphilum* enzymes (80**–**81% identities). On the contrary, DoeA of *Mm*. *alcaliphilum* exhibited only a 30**–**31% identity with DoeA from methanotrophs of the genus *Methylobacter* and from *Mh*. *crimeensis* (Fig 1). In addition, DoeA of *Mm*. *alcaliphilum* (phylum *Proteobacteria*) shared much higher similarity (~70% identity) with DoeA from *Tsukamurella paurometabola* (the phylum *Actinobacteria*) possessing the *doeBDAC* cluster than with DoeA from the proteobacterial *Rhodobacter sphaeroides* and *Nitrosococcus halophilus* harboring separately located genes of ectoine catabolism.

In *Mm*. *alcaliphilum*, the cluster of ectoine catabolic genes lacks the genes encoding the AsnC/Lrp-like transcriptional repressor and the MocR/GabR-type activator EnuR found in the *H*. *elongata* operon [6, 18]. The operon of *H*. *elongata* also possesses two genes involved in the conversion of hydroxyectoine into ectoine [6]. Though the sequence homologous to the ectoine hydroxylase encoding gene is present in the *Mm*. *alcaliphilum* genome, we failed to prove its functionality and hydroxyectoine accumulation in the methanotroph [30].

### Study of ectoine uptake by methanotrophic cells

The genetic determinants potentially responsible for the transport systems of ectoine were searched in *Mm*. *alcaliphilum* 20Z on the basis of sequences coding for the specific transporters of ectoine and hydroxyectoine in Gram-negative bacteria [18]. We have failed to find sequences homologous to the genes coding for the EhuABC transporter [19] in the genome of the methanotroph. The sequences slightly similar (15**–**20% identities) to the genes encoding hydroxyectoine/ectoine-specific Ueh TRAP transporter were found in the bacteria utilizing ectoines as nutrients [18] (S4 Table). Moreover, while growing in the medium with 5% NaCl and 1 mM ectoine, the cells of both the wide type strain 20Z and the strain Δ*ectBC* deficient in the ability to synthesize ectoine accumulated exogenous ectoine (Table 2). In the mutant strain Δ*ectBC*, intracellular ectoine content was twice lower than in the wild type strain. The cells of the strain with impaired *ectBC* genes had a higher level of sucrose (Table 3) thus balancing intracellular osmotic pressure. The addition of 1 mM trehalose to the growth media of both strains as a negative control had no effect on intracellular sucrose level.

**Table 2:** The intracellular accumulation of ectoine in the wild type cells of *Mm*. *alcaliphilum* 20Z and in strains ΔectBC1 with deleted ectoine biosynthesis genes. The cells were grown to the early stationary stage (OD_600_ = 2.1) at a salinity of 3%, 5% NaCl and 5% NaCl in the presence of 1 mM ectoine. Sucrose content was measured by HPLC as indicated in Materials & Methods.

Strain	Ectoine (mg/g of wet cells)		
	3% NaCl	5% NaCl	5% NaCl + 1 mM ectoine*
WT	6.3 ± 0.3	14.5 ± 0.7	19.3 ± 2.1
Δ*ectBC*	0.14 ± 0.02	0.12 ± 0.05	9.01 ± 0.81

*The residual ectoine concentration in the culture liquid of the wild type strain was 0.048 mM and that in the mutant strain Δ*ectBC* was 0.058 mM.

**Table 3:** The intracellular accumulation of sucrose in the wild type cells of *Mm*. *alcaliphilum* 20Z and in the strain with inactivated genes for ectoine biosynthesis. The cells were grown to the early stationary stage (OD_600_ = 2.1) at a salinity of 3% or 5% NaCl or at 5% NaCl in the presence of 1 mM ectoine or 1 mM trehalose. Sucrose content was measured by HPLC as indicated in Materials & Methods.

Strain	Sucrose (HPLC) (mg/g of wet cells)			
	3% NaCl	5% NaCl	5% NaCl + 1 mM ectoine	5% NaCl + 1 mM trehalose
WT	9.6 ± 0.6	11.2 ± 1.81	3.97 ± 0.39	11.09 ± 0.5
Δ*ectBC*	21.22 ± 1.37	35.94 ± 0.76	21.15 ± 0.83	30.16 ± 2.42

## Discussion

**Fig 4:**
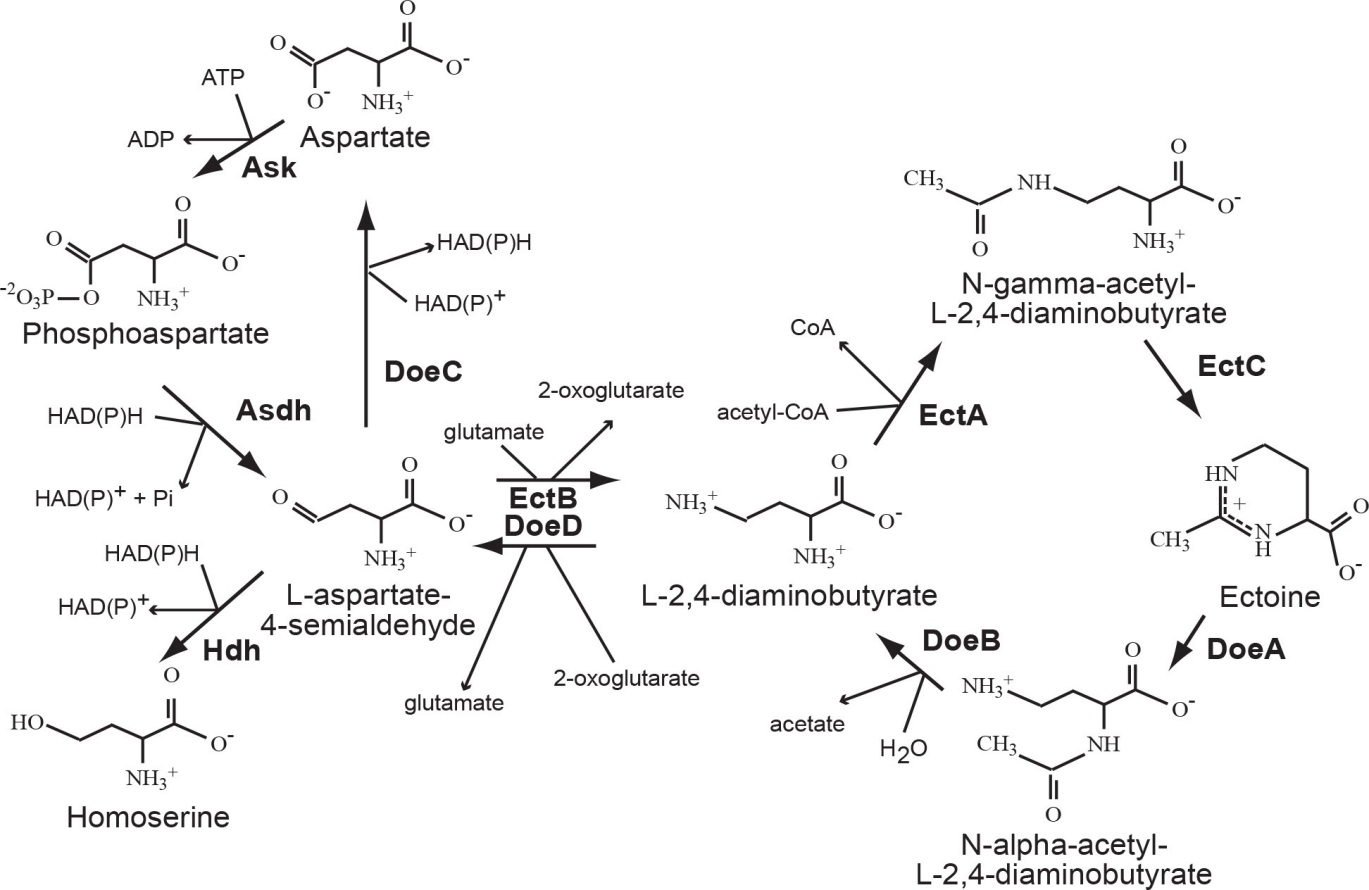
The metabolic pathway of ectoine in *Mm*. *alcaliphilum* 20Z. The scheme of the degradation pathway was constructed on the basis of the genetic and chromatographic analysis performed in this work. Ask: aspartate kinase; AsdH: b-aspartate-semialdehyde-dehydrogenase; Hdh: homoserine dehydrogenase; EctB: L-2,4-diaminobutyrate transaminase; EctA: L-2,4-diaminobutyrate Nγ-acetyltransferase; EctC: ectoine synthase; EctD: ectoine hydroxylase; DoeA: ectoine hydrolase; DoeB: Nα-acetyl-L-2,4-diaminobutyrate deacetylase; DoeD: L-2,4-diaminobutyrate transaminase; DoeC: aspartate-semialdehyde dehydrogenase.

Here we have shown for the first time that adaptation of the halotolerant methanotroph *Mm*. *alcaliphilum* 20Z to the changes in external osmolarity includes intracellular degradation of ectoine. In the methanotroph, four putative enzymes catalyzing ectoine catabolism (ectoine hydrolase, N-acetyl-DAB deacetylase, diaminobutyrate deaminase and aspartate-semialdehyde dehydrogenase) are encoded by the single operon *doeBDAC* (Figs 1 and 4). *In vivo* operation of at least two enzymes (ectoine hydrolase DoeA and N-acetyl-DAB deacetylase DoeB) was confirmed by mutational analysis. Disruption of the gene of ectoine hydrolase, the starting enzyme of the pathway, did not change the growth rate of the mutant strain, suggesting that the carbon flux through this pathway did not generate significant metabolic imbalance in the adapted culture. Nevertheless, the deletion of this gene led to an increase in the intracellular ectoine content. Only a slight increase in ectoine content in the mutant strain could be explained by the presence of a mechanism regulating ectoine biosynthesis via the transcriptional repressor EctR.

Our results demonstrate some distinctive features of ectoine metabolism in the obligate methanotroph and the previously studied heterotrophic bacteria. In *Mm*. *alcaliphilum*, Nα-acetyl-DAB is a major product of the ectoine hydrolase DoeA. In *H*. *elongata*, DoeA produced both isomers of N-acetyl-DAB. It is not improbable that Nγ-acetyl-DAB can also be formed during ectoine degradation in the methanotroph, but in low amounts that could not be identified due to the low activity of DoeA in both strain 20Z and recombinant *E*. *coli* cells. However, chromatographic analysis of cytoplasm of the *Mm*. *alcaliphilum* Δ*doeB* mutant and *E*. *coli* expressing methanotrophic doeA indicates that Nγ-acetyl-DAB is formed, if at all, in a much lower amount compared to the enzyme from *H*. *elongata* producing Nγ- and Nα-acetyl-DAB at the ratio of 1:2. If this ratio is true for the enzymes of both bacteria, we could detect Nγ-acetyl-DAB in our experiments. In *H*. *elongata*, N-γ-acetyl-DAB, the substrate of ectoine synthase EctC, is also an inducer of the EnuR protein, which controls transcription of the ectoine/hydroxyectoine biosynthetic pathway [20]. Diaminobutyric acid in this heterotrophic bacterium can either flow off to aspartate or re-enter the ectoine synthesis pathway, forming a cycle of ectoine synthesis and degradation. In the genome of the methanotroph, we did not find any sequences for the EnuR-like genes and the genes encoding the AsnC/Lrp-like transcriptional repressor. In our experiments, Nα-acetyl-DAB (a substrate for DoeB) was detected in response to osmotic down-shock of Δ*doeB* cells, thus indicating the non-constitutive character of ectoine catabolism in the methanotroph. Disappearance of the Nγ-acetyl-DAB peak from the cells of the ΔdoeB strain exposed to osmotic down-shock also implies that the ectoine synthase (EctC) could remain functional under these conditions when the ectione degradation pathway operates. Moreover, the intracellular concentration of ectoine did not always clearly correspond to the osmotic pressure of the medium and significantly increased under certain conditions (i.e., in the presence of ectoine in the medium or overexpression of the enzymes involved in ectoine synthesis). We believe that the increase in intracellular osmolarity due to the excess of ectoine does not initiate the degradation mechanism, since it could be compensated by changes in the levels of other metabolites (sucrose). It is also interesting that ectoine degradation was activated upon a mild dilution stress, indicating that cells preserve the previously invested carbon and nitrogen resources via ectoine synthesis for metabolism under the conditions when very high cellular pool of ectoine is no longer needed. *Mm*. *alcaliphilum* is an obligate C1-utilizer incapable of using any organic compound as a single growth substrate; nevertheless, acetate and aspartate, the products of the ectoine degradation route, can enter the central biochemical pathways in the methanotroph. Acetate can enter the tricarboxylic acid (TCA) cycle after the activation by acetate kinase [31]. In addition, the oxaloacetate derived from aspartate can be reduced to malate, which is subsequently converted to pyruvate via the malic enzyme [32]. The special role of the NAD^+^-malic enzyme in the withdrawal of excessive C4 intermediates from the TCA cycle has been proposed recently [32].

Since no ectoine was found in the growth medium after fourfold dilution of the culture (from 6% NaCl to 1.5% NaCl), we can assume the absence of ectoine extrusion from cells, at least under the conditions tested. The genome sequence analysis showed that *Mm*. *alcaliphilum* codes for at least four proteins (WP_014150036, WP_014148079, WP_014150313, WP 014148079) with identities to MscL/MscS mechanosensitive channel proteins from *E*. *coli*. The proteins of MscS family make water-filled open channel pores of ~13–16 Å in diameter [33]. The MscL proteins make very large pores in the membrane that are ∼30 Å in diameter (the largest gated pore known) and, if this channel is gated inappropriately, its presence is devastating for the cell [34]. An absence of ectoine in the medium indicates that moderate osmotic down-shock does not initiate the pore opening (by MscL homolog) in the methanotroph, that would be sufficient for ectoine excretion, and the pores created by the MscS family proteins are too small.

Here we have shown that *Mm*. *alcaliphilum* is able to take up ectoine from the medium simultaneously with synthesis of osmoprotectant. The candidate for the respective transporter is the TRAP-type transporter, which is considered to use electrochemical sodium gradients to energize this consumption. However, we did not estimate the affinity and specificity of these systems to ectoine. In contrast to many Gram-negative bacteria utilizing ectoine as a growth substrate, the genes encoding putative solute transporters in methylotrophs are located outside the cluster of ectoine catabolism. We have also shown that the cytoplasmic glutamate content increases upon the increase in salinity of the medium, thus confirming the osmoprotective role of the amino acid [1]. However, no noticeable correlation between the glutamate and ectoine contents in the mutant strains has been detected.

Although ectoine was first detected in the extremely halophilic phototrophic bacterium *Ectothiorhodospira halochloris* [1], the metabolism of this osmoprotector has been studied mainly in heterotrophic microorganisms. In this work we have considered for the first time some aspects of ectoine catabolism in bacteria utilizing only C1 compounds as carbon sources. The differences in ectoine degradation pathways in heterotrophs and obligate methylotrophs are apparently related to their physiological traits. Heterotrophic bacteria have developed a more complex regulatory mechanism, which allows them to switch from the use of ectoine as an osmoprotector to its use as a substrate for growth. In methanotrophs, the function of ectoine is predominantly associated with osmoadaptation and the pathway of ectoine degradation serves for preservation of carbon, nitrogen and energy accumulated in the osmoprotector molecule. This correlates with the differences in genetic organization of the ectoine degradation pathway in methylotrophs and heterotrophs.

## Supporting information

S1 Fig The chromatograms of intracellular solutes in the wild type strain of *Mm*. *alcaliphilum* 20Z in the Reprosil OPA column with o-phthalaldehyde (OPA) pre-column derivatization. A, the glutamate standard; B, the standards of Nγ-acetyl-DAB and Nα-acetyl-DAB obtained by the alkaline hydrolysis of pure ectoine [29]. The culture was grown under methane in a mineral salt medium in the presence of 6% NaCl (C) diluted with the medium without NaCl to a final concentration of 1.5% NaCl (D, E) and incubated for 40 min (D) or 90 min (E) under the optimal growth conditions.
(PDF)

S2 Fig The phylogenetic tree of the putative DoeA proteins from methylotrophic bacteria and DoeA from heterotrophic bacteria with the established ectoine-degradation pathway. (PDF)

S1 Table Primers used in this study. (DOCX)

S2 Table Bacterial strains and plasmids used in this study. (DOCX)

S3 Table The putative proteins encoding by *doeBDAC* gene cluster detected in the genome of *Mm*. *alcaliphilum* 20Z. (DOCX)

S4 Table Sequences encoding putative solute transporters found in genomes of *Methylomicrobium alcaliphilum* 20Z and their identity (%) to the respective genes of the 5-hydroxyectoine/ectoine TRAP transporters found in the *Ruegeria pomeroyi* DSS-3 [7] and *Halomonas elongata* DSM 2581 [8]. (DOCX)
