# Supplementary figures and images for: Ectoine degradation pathway in halotolerant methylotrophs
Source: PLoS One. 2020 Apr 30;15(4):e0232244. doi: 10.1371/journal.pone.0232244 (PMC7192451; doi:10.1371/journal.pone.0232244)

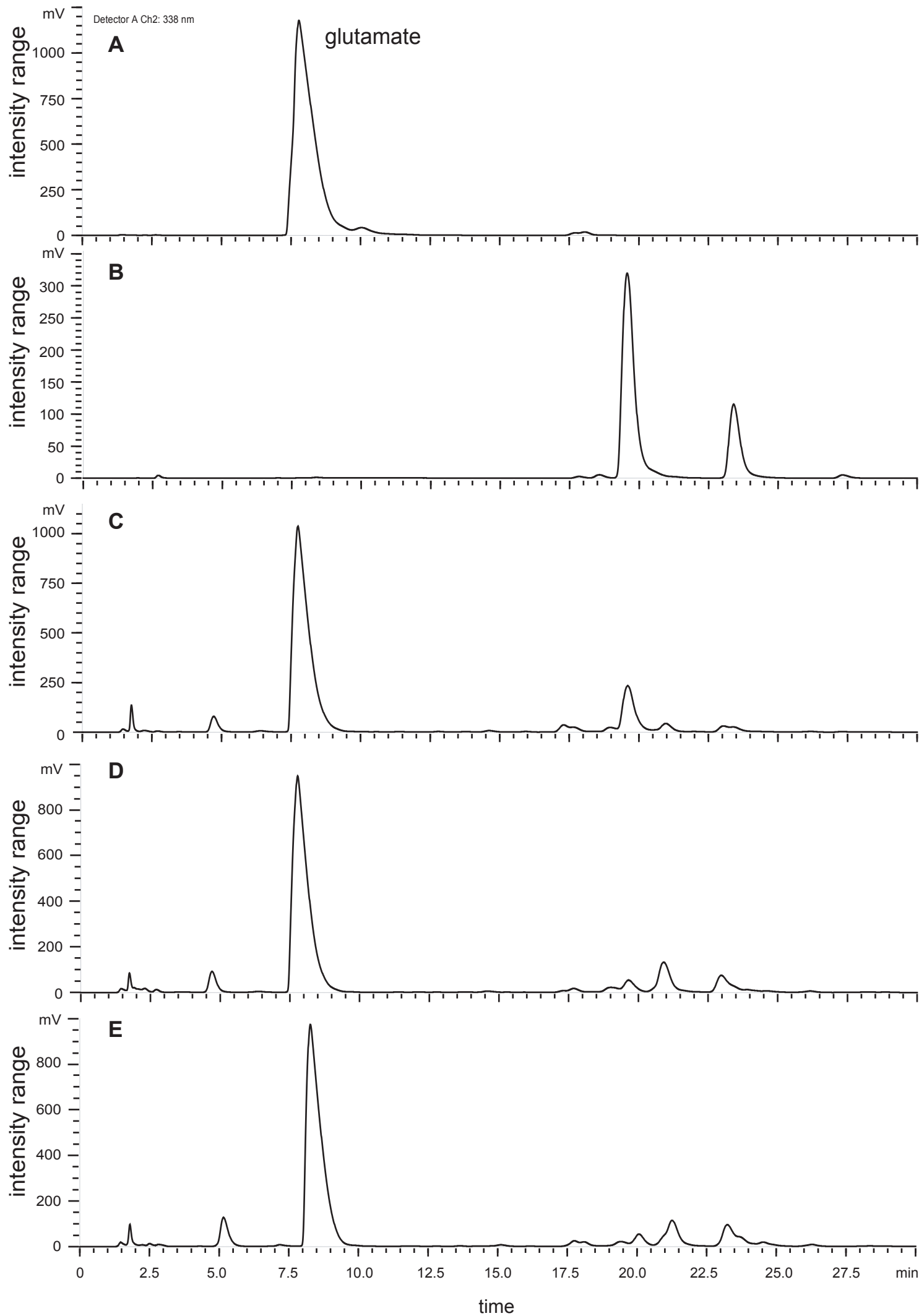

Fig. S1

Supplement: S1 Fig — A, the glutamate standard; B, the standards of Nγ-acetyl-DAB and Nα-acetyl-DAB obtained by the alkaline hydrolysis of pure ectoine [29]. The culture was grown under methane in a mineral salt medium in the presence of 6% NaCl (C) diluted with the medium without NaCl to a final concentration of 1.5% NaCl (D, E) and incubated for 40 min (D) or 90 min (E) under the optimal growth conditions. (PDF) [file pone.0232244.s001.pdf]

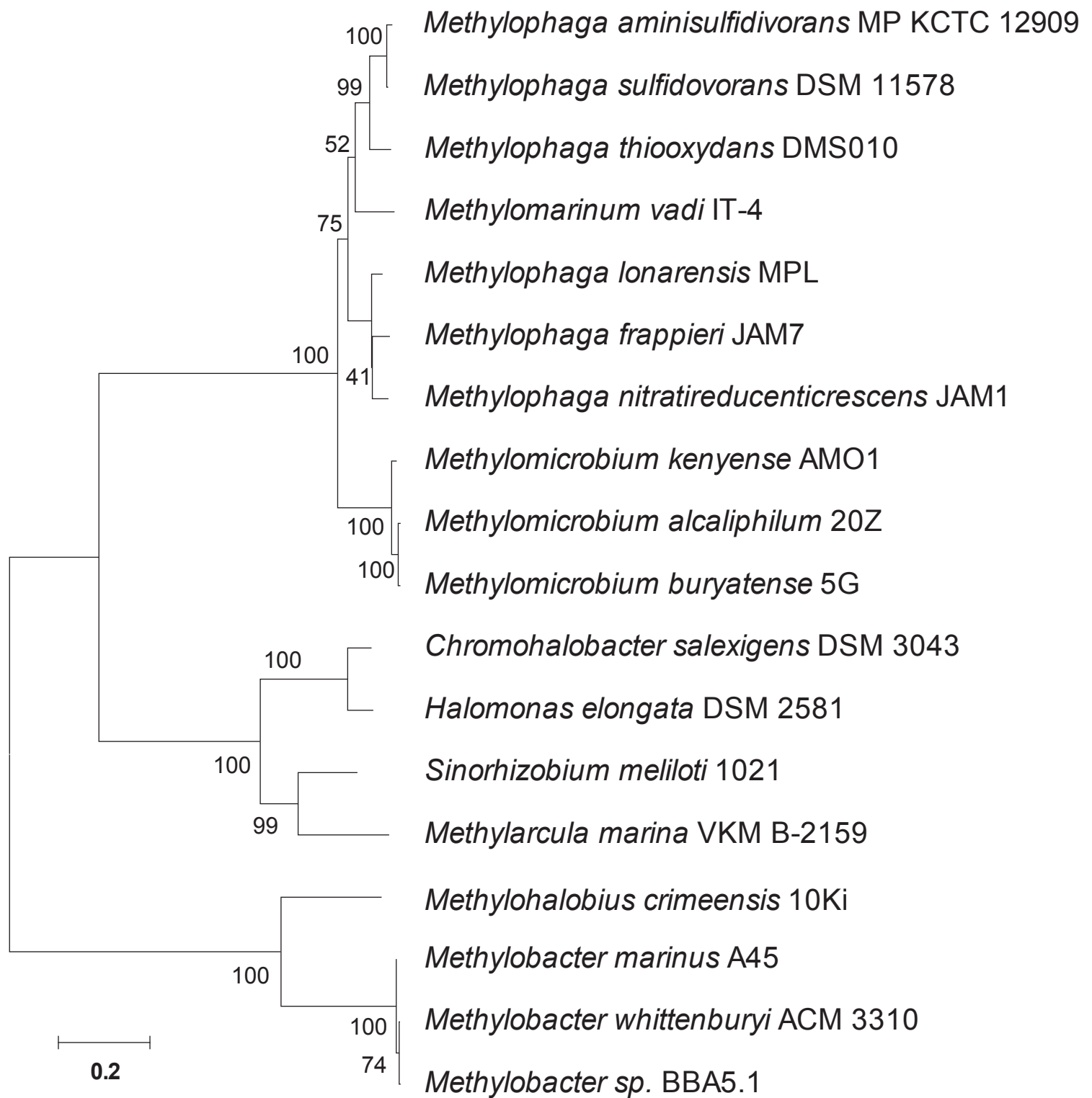

Fig. S2

Supplement: S2 Fig — (PDF) [file pone.0232244.s002.pdf]
