# Supplementary material for: Ectoine degradation pathway in halotolerant methylotrophs
Source: PLoS One. 2020 Apr 30;15(4):e0232244. doi: 10.1371/journal.pone.0232244 (PMC7192451; doi:10.1371/journal.pone.0232244)
Supplement: S1 Table — (DOCX) [file pone.0232244.s003.docx]

**Table S1. Primers used in this study**

| **Primer name** | **Sequence (5′ to 3′)^a^** | **Application or target** |
| --- | --- | --- |
| DoeAF  DoeAR | AACATATGATTGAACGCGACGACA  TTAAGCTTAGCCCCGTATTCGGGT | *doeA* gene |
| DoeBF  DoeBR | AACATATGGCCATACAGTGGGATCA  TTAAGCTTTGCTCGTTCCTTAACGTCA | *doeB* gene |
| doeAmutF1  doeAmutR1 | TTAGATCTGCATGGATTTGGAACCGGACA  TTGGTACCAGTTCGTCGATTCGGCGTT | upstream and 5′-fragment of *doeA* for deletion of the gene |
| doeAmutF2  doeAmutR2 | TACCGCGGACGAAGGTTACATCATCA  TTGAGCTCTCCAATCGGCATAGG | downstream and 3′-fragment of *doeA* for deletion of the gene |
| doeBmutF 1  doeBmutR1 | ATAGATCTATGGAGTAGTTCAAAATTCGGC  TTGGTACCGGACATGACGGCCACA | upstream and 5′-fragment of *doeB* for deletion of the gene |
| doeBmutF 2  doeBmutR2 | TTGGGCCCTTTCGCTTTAATCGGCAC  TTGAGCTCTGTTGTGGCCGAAATTGAGTAC | downstream and 3′-fragment of *doeB* for deletion of the gene |
| DoeA(HSG575)  DoeA(HSGstop) | TTGGATCCTGATGGAGGAGATATTATGATTGAACGCGACGACATGAC  TCAAGCTTTCAAGCCCCGTATTCGGGTTTC | for cloning of the *doeA* in pHSG575 |
| ectABC_F(Sac)  ectABC_R(Vsp) | AGAGCTCAGGAGTATTTATATGTTGCCTGAT  AATTAATCCTCTCTTACTTCGTGGAT | for cloning of *ectABC* operon |

^a^ Restriction enzyme recognition sites are underlined
