# Supplementary material for: Ectoine degradation pathway in halotolerant methylotrophs
Source: PLoS One. 2020 Apr 30;15(4):e0232244. doi: 10.1371/journal.pone.0232244 (PMC7192451; doi:10.1371/journal.pone.0232244)
Supplement: S2 Table — (DOCX) [file pone.0232244.s004.docx]

**Table S2.**

Bacterial strains and plasmids used in this study

| **Strains and plasmids** | **Description** | **Reference or source** |
| --- | --- | --- |
| *Escherichia coli* | | |
| S17-1 | *pro, res^−^ hsdR17 (rK^−^ mK^+^) recA^−^* with an integrated *RP4-2-Tc::Mu-Km::Tn7*, Tp^r^ | [1] |
| Rosetta (DE3) | F^-^ *ompT hsdS*_B_(r_B_^-^ m_B_^-^) *gal dcm* (DE3) pRARE (Cam^R^) | Novagen |
| DH5α | F^-^ φ*80dlacZDM15* Δ(*lacZYA-argF*) *U169 recA1 hsdR17* (*rK^-^ mK^+^*) *supE44* Δ^-^ *thi-1 gyrA relA1* | [2] |
| *Methylomicrobium alcaliphilum* | | |
| 20Z | wild type | [3] |
| ΔdoeA | *doeA* deletion mutant | This study |
| ΔdoeB | *doeB* deletion mutant | This study |
| ΔdoeA pMHA300_Pmxa_ectABC | *doeA* deletion mutant transformed by pMHA300_Pmxa_ectABC plasmid | This study |
| Plasmids | | |
| pET22b | Bacterial vector for inducible expression of proteins | Invitrogen |
| pET22b_ *doeA* | pET22b carrying *doeA* gene | This study |
| pET22b_ *doeB* | pET22b carrying *doeB* gene | This study |
| pHSG575 | pSC101 replicon, *lacZ*α+ Cm^R^ | [4] |
| pHSG575_doeA | pHSG575 carrying *doeA* gene | This study |
| pCM184 | Ap^R^, Km^R^, Tc^R^; broad-host range *cre-lox*allelic exchange vector | [5] |
| pCM184doeA | pCM184 carrying 3′- and 5′- fragments of *doeA* gene upstream and downstream the Km gene | This study |
| pCM184doeB | pCM184 carrying 3′- and 5′- fragments of *doeB* gene upstream and downstream the Km gene | This study |
| pMHA200_Pmxa_cat | pMHA200, with *cat* gene under control the Pmxa promoter | [6] |
| pMHA200_Pmxa_ectABC | In pMHA200_Pmxa_cat the *cat* gene was replaced by *ectABC* operon from *Methylomicrobium alcaliphilum* 20Z | This study |
| pMHA300_Pmxa_ectABC | The Km gene in pMHA200_Pmxa_ectABC was replaced by Gm gene | This study |

**References:**

1. Simon R, Priefer U, Puhler A. A broad host range mobilization system for in vivo genetic-engineering - transposon mutagenesis in gram-negative bacteria. Bio-Technol. 1983; 1:784–791.
2. Hanahan D. Studies on transformation of *Escherichia coli* with plasmids. J Mol Biol 1983; 166:557–580.
3. Khmelenina VN, Kalyuzhnaya M, Sacharovski VG, Suzina NE, Trotsenko YA, Gottschalk G. Osmoadaptation in halophilic and alkaliphilic methanotrophs. Arch Microbiol. 1999; 172:321-329.
4. Takeshita S, Sato M, Toba M, Masahashi W, Hashimoto-Gotoh T.  High-copy-number and low-copy-number vectors for *lacZα*-complementation and chloramphenicol- or kanamycin-resistance selection.  Gene. 1987; 61:63–74.
5. Marx CJ, Lidstrom ME. Broad-host-range *cre-lox* system for antibiotic marker recycling in gram-negative bacteria. Bio Techniques. 2002; 33:1062–1067.
6. Mustakhimov II, But SY, Reshetnikov AS, Khmelenina VN, Trotsenko YA. Homo- and heterologous reporter proteins for evaluation of promoter activity in *Methylomicrobium alcaliphilum* 20Z. Prikl Biokhim Mikrobiol (Russian). 2016; 52:279–286.
7. Schulz A, Stöveken N, Binzen IM, Hoffmann T, Heider J, Bremer E. Feeding on compatible solutes: A substrate-induced pathway for uptake and catabolism of ectoines and its genetic control by EnuR. Environ Microbiol. 2017; 19(3):926-946.
8. Grammann K, Volke A, Kunte HJ. New type of osmoregulated solute transporter identified in halophilic members of the bacteria domain: TRAP transporter TeaABC mediates uptake of ectoine and hydroxyectoine in *Halomonas elongata* DSM 2581(T). J Bacteriol. 2002; 184:3078-3085.
