# Supplementary material for: Ectoine degradation pathway in halotolerant methylotrophs
Source: PLoS One. 2020 Apr 30;15(4):e0232244. doi: 10.1371/journal.pone.0232244 (PMC7192451; doi:10.1371/journal.pone.0232244)
Supplement: S3 Table — (DOCX) [file pone.0232244.s005.docx]

**Table S3** The putative proteins encoding by *doeBDAC* gene cluster detected in the genome of *Mm. alcaliphilum* 20Z

| ORF | Number of amino acids | kDa | Most probable coding protein | Identity with the  *H. elongata* protein |
| --- | --- | --- | --- | --- |
| MEALZ_3978 | 391 | 43.9 kDa | DoeA, ectoine hydrolase | 42% |
| MEALZ_3976 | 393 | 42.6 kDa | DoeB, ectoine deacetylase | 22% |
| MEALZ_3977 | 425 | 46.2-kDa | DoeD, transaminase | 36% |
| MEALZ_3979 | 453 | 48.5 kDa | DoeC, aspartate-semialdehyde dehydrogenase | 49% |
