# Supplementary material for: Ectoine degradation pathway in halotolerant methylotrophs
Source: PLoS One. 2020 Apr 30;15(4):e0232244. doi: 10.1371/journal.pone.0232244 (PMC7192451; doi:10.1371/journal.pone.0232244)
Supplement: S4 Table — (DOCX) [file pone.0232244.s006.docx]

**Table S4** Sequences encoding putative solute transporters found in genomes of *Methylomicrobium alcaliphilum* 20Z and their identity (%) to the respective genes of the 5-hydroxyectoine/ectoine TRAP transporters found in the *Ruegeria pomeroyi* DSS-3 [7] and *Halomonas elongata* DSM 2581 [8].

|  | ***Ruegeria pomeroyi*** DSS-3 | | | ***Halomonas elongata* DSM 2581** | | |
| --- | --- | --- | --- | --- | --- | --- |
|  | **large integral membrane protein (UehC)**  [AAV94447.1](https://www.ncbi.nlm.nih.gov/protein/56677781) | **small integral membrane protein (UehB)**  [AAV94446.1](https://www.ncbi.nlm.nih.gov/protein/56677781) | **periplasmic binding protein (UehA)**  [AAV94445.1](https://www.ncbi.nlm.nih.gov/protein/56677781) | **ectoine-binding periplasmic protein (TeaA)**  [WP_013334028.1](https://www.ncbi.nlm.nih.gov/protein/503099229) | **small permease (Tea B)**  [WP_013334029.1](https://www.ncbi.nlm.nih.gov/protein/503099230) | **large transmembrane protein (TeaC)**  [WP_013334030.](https://www.ncbi.nlm.nih.gov/protein/503099231) |
| TRAP dicarboxylate transporter-DctM subunit [CCE23287.1](https://www.ncbi.nlm.nih.gov/protein/351717622) |  |  | 29.1 |  |  | 29.1 |
| TRAP C4-dicarboxylate transporter  [CCE23288.1](https://www.ncbi.nlm.nih.gov/protein/351717622) |  | 19.7 |  |  | 19.2 |  |
| Extracellular solute-binding protein, family [CCE23289.1](https://www.ncbi.nlm.nih.gov/protein/351717622) | 15.5 |  |  | 15.8 |  |  |
| Putative TRAP C4-dicarboxylate transport system, DctQ subunit [CCE22928.1](https://www.ncbi.nlm.nih.gov/protein/351717263) |  | 14.0 |  |  | 16.8 |  |
| Putative TRAP dicarboxylate transporter, DctM subunit [CCE22927.1](https://www.ncbi.nlm.nih.gov/protein/351717262) |  |  | 26.5 |  |  | 25.8 |
